# Soliton driven angiogenesis

**DOI:** 10.1038/srep31296

**Published:** 2016-08-09

**Authors:** L. L. Bonilla, M. Carretero, F. Terragni, B. Birnir

**Affiliations:** 1 Gregorio Millán Institute for Fluid Dynamics, Nanoscience and Industrial Mathematics and Department of Materials Science & Engineering, Universidad Carlos III de Madrid, Avenida de la Universidad 30, 28911 Leganés, Spain; 2 Center for Complex and Nonlinear Science and Department of Mathematics, University of California at Santa Barbara, USA

## Abstract

Angiogenesis is a multiscale process by which blood vessels grow from existing ones and carry oxygen to distant organs. Angiogenesis is essential for normal organ growth and wounded tissue repair but it may also be induced by tumours to amplify their own growth. Mathematical and computational models contribute to understanding angiogenesis and developing anti-angiogenic drugs, but most work only involves numerical simulations and analysis has lagged. A recent stochastic model of tumour-induced angiogenesis including blood vessel branching, elongation, and anastomosis captures some of its intrinsic multiscale structures, yet allows one to extract a deterministic integropartial differential description of the vessel tip density. Here we find that the latter advances chemotactically towards the tumour driven by a soliton (similar to the famous Korteweg-de Vries soliton) whose shape and velocity change slowly. Analysing these collective coordinates paves the way for controlling angiogenesis through the soliton, the engine that drives this process.

Angiogenesis is a multiscale process spanning scales from subcellular to millimetre ones by which blood vessels grow from existing ones and carry oxygen to distant organs^1^,^2^,^3^. Angiogenesis is essential for normal growth of organs in embryos and repair of wounded tissue in adults. Angiogenesis imbalance may lead to malignant, ocular and inflammatory disorders, and it affects asthma, diabetes, cirrhosis, AIDS, ischemic heart disease, multiple sclerosis and autoimmune diseases among others^1^. In recent years, understanding of the molecular mechanisms of angiogenesis has increased at an explosive rate and has led to the approval of anti-angiogenic drugs for cancer and eye diseases^4^. Combined with experiments, mathematical and computational models contribute substantially to these efforts; see ref. 5 for a state of the art review. Models range from those capturing cell dynamics at cellular scale^6^,^7^,^8^ to mesoscopic endothelial cell migration models that do not describe the cellular scale^9^,^10^,^11^,^12^,^13^,^14^,^15^,^16^,^17^,^18^,^19^,^20^.

Most work has dealt with numerical solutions of models and their analysis has lagged behind. In this work, we consider a recent stochastic model of tumour-driven angiogenesis including tip branching, elongation, and anastomosis of blood vessels (simulated in Fig. 1 and sketched in fig. 2) that has been shown to capture some of the intrinsic multiscale structures of this complex system^19^,^21^,^22^. The vessel network is the set of all trajectories of tip cells (blood vessels are thus assumed to follow the paths of tip cells), **X**^*i*^(*t*), *i* = 1, …, *N*(*t*), that move with velocities **v**^*i*^(*t*). Elongations of tips are described by Ito stochastic differential equations (**W**^*i*^(*t*) are independent identically distributed Brownian motions) whereas tip branching and anastomosis are birth and death processes that change the number of active tips. While it is standard to obtain a deterministic description of a tip density based on Ito equations^23^, a recent breakthrough has resulted in including the effect of vessel fusion (anastomosis) in the deterministic description for the tip density^21^,^22^. This counterpart deterministic description is also shown in Fig. 2. The vessel tip density is a mean over many realisations or replicas of the stochastic process (ensemble average)^22^, and it is the unique solution of a system of integropartial differential equations^24^. During tumour induced angiogenesis, the marginal tip density, 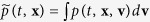, forms a lump that grows and moves towards the tumour, as shown in Fig. 3. The lump profile, 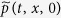, is that of a moving pulse. By analyzing the deterministic equations and simulating both them and the stochastic model, we show here that this pulse is approximately a soliton similar to that of the famous Korteweg-de Vries equation for water waves^25^. Angiogenesis is driven by this soliton which, in turn, is determined by two parameters or collective coordinates. The latter respond to transport processes such as chemotaxis or diffusion that are affected by e.g. anti-angiogenic treatments. This paves the way to controlling a complex multiscale biological process by controlling the much simpler description provided by the soliton collective coordinates.

## Results

**Figure 2:**
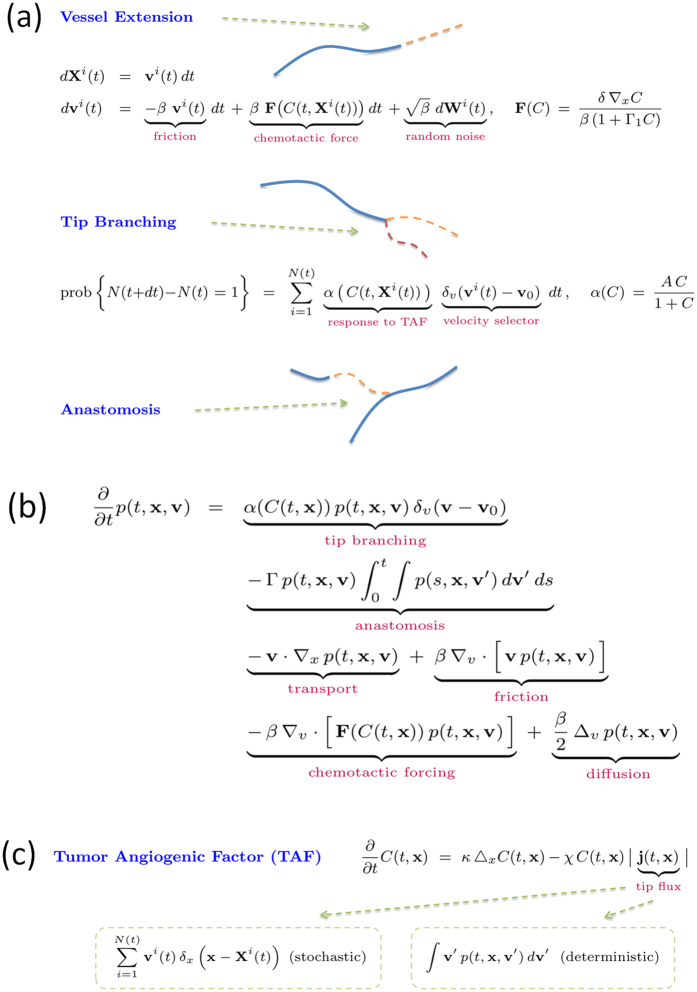
(**a**) Stochastic model of tumour induced angiogenesis comprising vessel extension, tip branching and anastomosis. (**b**) Deterministic description for the vessel tip density 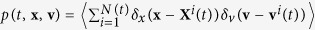. (**c**) Equation for the TAF density. *δ*_*x*_(**x**) and *δ*_*v*_(**v**) are Gaussian regularizations of delta functions and all equations are written in nondimensional units^22^.

**Figure 4:**
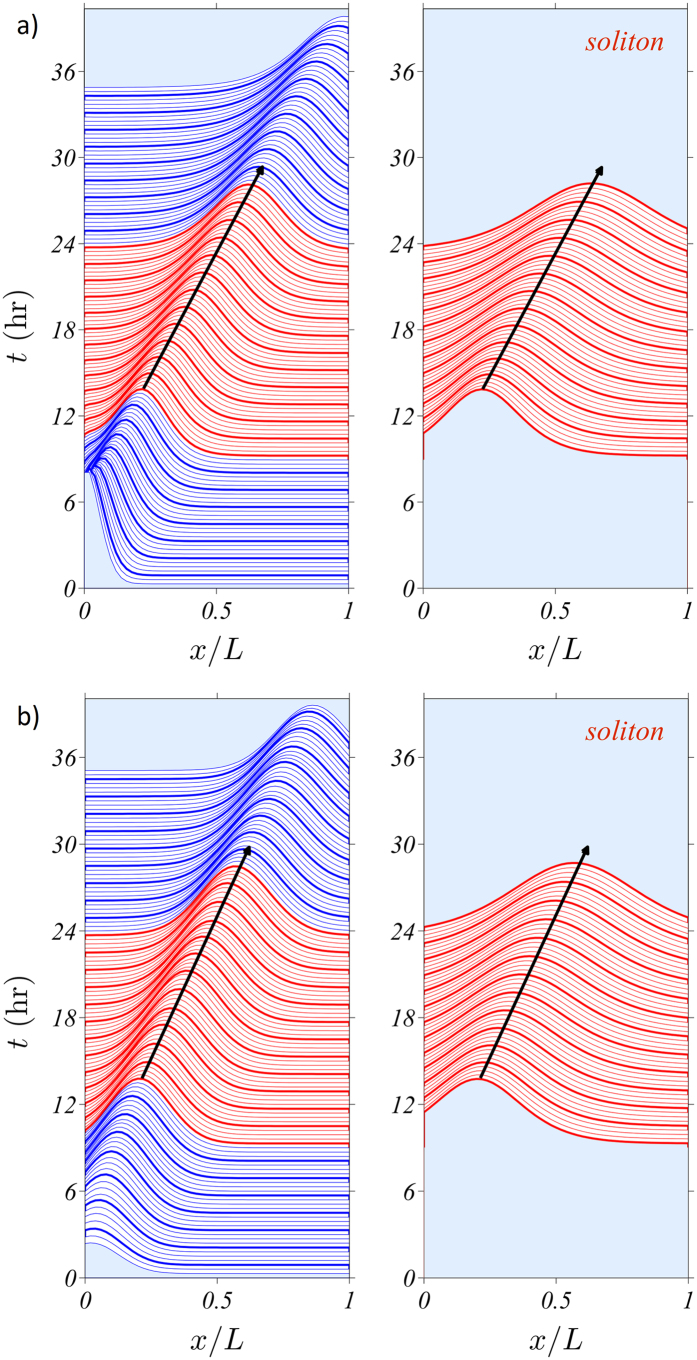
Comparison of the marginal tip density profile to that of the moving soliton. (**a**) Continuum description. (**b**) Stochastic description averaged over 400 replicas.

Except for an initial stage of detachment from the primary vessel and a final stage of arrival at the tumour, the tip density profile is close to a soliton similar to the Korteweg-de Vries soliton; see Fig. 4. To see why this is so, we consider the overdamped limit of stochastic vessel extension in Fig. 2(a), 
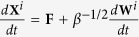
, and write the corresponding deterministic equation for the marginal tip density as


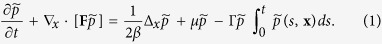


Here the chemotactic force **F** and the renormalized tip branching rate *μ* are known functions of the tumour angiogenic factor *C*(*t*, **x**). Provided *β* is large, *C* is slowly-varying and **F** is predominantly aligned along the *x* axis, the previous equation may be approximated by


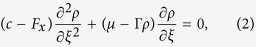


where *F*_*x*_ is the *x* component of **F**, and 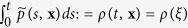 with 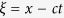. We solve this equation for *ρ* and determine 
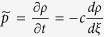
, with the result


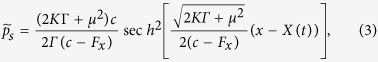


for the soliton. Here *X*(*t*) = *ct* + *ξ*_0_ and *K* and *ξ*_0_ are constants. This expression resembles the Korteweg-de Vries soliton^25^. The soliton shape and velocity are determined by *K* and *c* and its position by *X*(*t*) such that 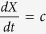. As we recall the small diffusion term in (1) and that the coefficients **F** and *μ* in that equation vary with *C*(*t*, **x**), we may surmise that *K* and *c* are collective coordinates whose change describes how the soliton advances towards the tumour. In the Methods section, we write the equations for the collective coordinates *K*(*t*) and *c*(*t*) corresponding to a soliton far from both the primary vessel and the tumour.

Our numerical simulations show that the vessel tip density approaches the soliton after some time. Initially there are few tips, the density is small and anastomosis is scarce. Tips branch and multiply, and anastomosis kicks in. The soliton formation should be described as the solution of a semi-infinite initial-boundary value problem. After the soliton (3) is formed, its evolution is governed by the collective coordinate equations (8)–(10) (Methods section). Figure 4 shows that the soliton approximates quite well both the solution of the deterministic description and the ensemble averaged vessel tip density for most of the vessel network evolution: after an initial stage of soliton formation and before the tip cells arrive at the tumour. What is most important is that angiogenesis is driven by soliton formation and motion. Including other mechanisms in our stochastic model such as haptotaxis through continuum fields providing extra forces representing e.g. fibronectin and matrix degrading enzymes can be done as indicated in other tip motion models^10^,^12^,^19^. These new fields affect soliton motion in ways similar to the growth factor and chemotaxis. Thus they can be included in our study with little changes affecting the collective coordinates only. Haptotaxis models that describe changes in cell shape, degradation of the extra cellular matrix, etc via cellular Potts models^6^,^8^ require additional studies to ascertain the effects of these microscopic processes on the mesoscopic scale described by tip or stalk cell density equations. Insofar as anti or pro-angiogenic treatments can be included in equations for the continuum fields^12^,^13^,^14^, their effect on the soliton can be ascertained and control of angiogenic sprouts may be reduced to a simpler problem of controlling the equations for the collective coordinates.

In conclusion, we have explained for the first time tumour induced angiogenesis as being driven by a soliton wave of the vessel tip density. After an initial stage, a lump in the tip density forms and its profile becomes that of a soliton whose shape and velocity are determined by diffusion of vessel tips and by the tumour angiogenic factor through the evolution of collective coordinates. Although the tip density appears as an ensemble average over many realisations of the stochastic process, the soliton velocity and position describe well that of any single replica. This opens a path to control angiogenesis through controlling the soliton, the engine that drives angiogenesis.

## Methods

### Equation for the marginal tip density

Equation (1) is derived by using the Chapman-Enskog method^26^ to approximate the solution of the deterministic description for the vessel tip density. We assume that


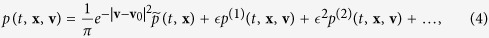



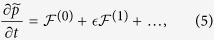


in which 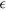 is a scaling parameter that we also insert in the equation for the tip density:


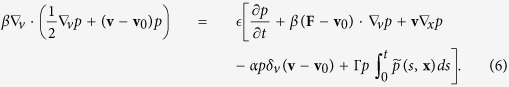


We have replaced *β***F** instead of **F** in the equations of ref. 22. Substituting (4) in (6) and taking into account (5), we obtain a hierarchy of equations in the limit of small 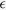. We determine the 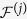 for *j* = 0, 1 such that *p*^(1)^ and *p*^(2)^ are bounded. The result is (1) with *μ* = *α*/*π* + *O*(1/*β*) once we set the scaling parameter 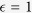.

### Collective coordinates

To find evolution equations for them and following ref. 27, we insert the soliton (3) in (1), thereby obtaining


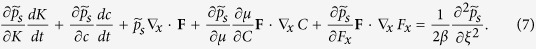


We now multiply (7) by 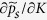 and integrate from *ξ* = −∞ to ∞. Then we multiply (7) by 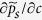 and integrate from *ξ* = −∞ to ∞. From the two resulting equations, we find the following system of ordinary differential equations for the collective coordinates


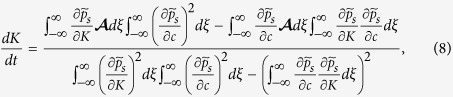



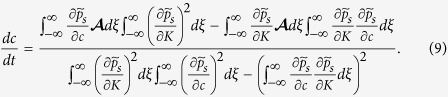



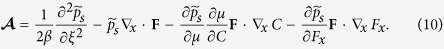


The integrals can be explicitly done by using Mathematica.

### Coefficients in the collective coordinate equations

The coefficients in these equations depend on the TAF concentration *C*(*t*, **x**) which is supposed to be almost constant. We calculate these constant values by setting *y* = 0 and averaging the resulting coefficients from *x* = 0 to 0.6. At larger values of *x*, the boundary condition at *x* = 1 influences the outcome. In our numerical simulations, we have used the same numerical values of the parameters as in ref. 22. The anastomosis coefficient Γ is found by fitting deterministic and stochastic simulations so that the total number of vessel tips is approximately the same; see ref. 22. The upper panels of Fig. 4 are produced by numerically solving the deterministic description and comparing the results to the solutions of the collective coordinate equations (8)–(9). The lower panels of Fig. 4 are produced by ensemble averages of stochastic simulations that are compared to the solutions of (8)–(9) with a fitted anastomosis coefficient.

## Additional Information

**How to cite this article**: Bonilla, L. L. *et al*. Soliton driven angiogenesis. *Sci. Rep*. **6**, 31296; doi: 10.1038/srep31296 (2016).

## Figures and Tables

**Figure 1:**
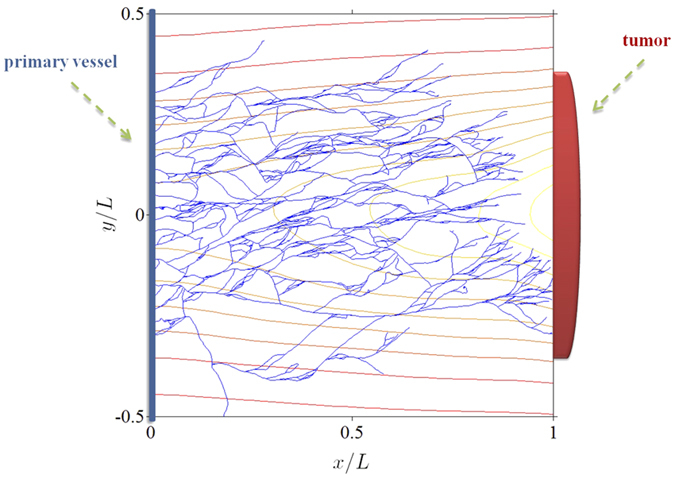
Network of blood vessels simulated by the stochastic model of tumour induced angiogenesis. The level curves of the density of the tumour angiogenic factor (vessel endothelial growth factor) are also depicted.

**Figure 3:**
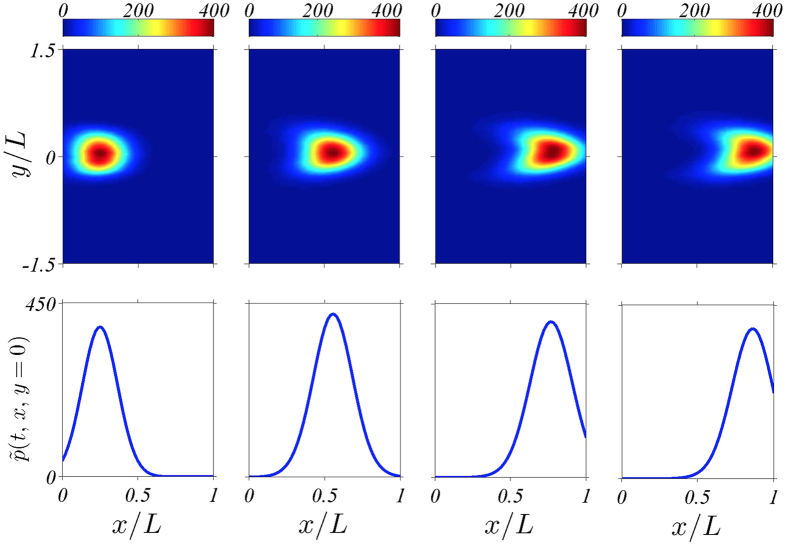
(**a**) Density plot of the marginal tip density 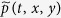 at different times showing how tips are created at the primary blood vessel at *x* = 0 and march towards the tumour at *x* = *L*. (**b**) Marginal tip density at *y* = 0 for the same times as in panel (**a**). The tip density has been calculated as an ensemble average over 400 replicas of the stochastic model.
